# Sensorimotor Control of Tracking Movements at Various Speeds for Stroke Patients as Well as Age-Matched and Young Healthy Subjects

**DOI:** 10.1371/journal.pone.0128328

**Published:** 2015-06-01

**Authors:** Di Ao, Rong Song, Kai-yu Tong

**Affiliations:** 1 School of Engineering, Sun Yat-sen University, Guangzhou, Guang Dong, P. R. China; 2 Division of Biomedical Engineering, Department of Electronic Engineering, the Chinese University of Hong Kong, Hong Kong, China; 3 Key Laboratory of Sensing Technology and Biomedical Instrument of GuangDong province, Guangzhou, Guang Dong, P. R. China; Georgia State University, UNITED STATES

## Abstract

There are aging- and stroke-induced changes on sensorimotor control in daily activities, but their mechanisms have not been well investigated. This study explored speed-, aging-, and stroke-induced changes on sensorimotor control. Eleven stroke patients (affected sides and unaffected sides) and 20 control subjects (10 young and 10 age-matched individuals) were enrolled to perform elbow tracking tasks using sinusoidal trajectories, which included 6 target speeds (15.7, 31.4, 47.1, 62.8, 78.5, and 94.2 deg/s). The actual elbow angle was recorded and displayed on a screen as visual feedback, and three indicators, the root mean square error (RMSE), normalized integrated jerk (NIJ) and integral of the power spectrum density of normalized speed (IPNS), were used to investigate the strategy of sensorimotor control. Both NIJ and IPNS had significant differences among the four groups (P<0.01), and the values were ranked in the following order: young controls < age-matched controls <unaffected sides of stroke patients <affected sides of stroke patients, which could be explained by the stroke- and aging-induced increase in reliance on feedback control. The RMSE increased with the increase in the target speed and the NIJ and IPNS initially declined and then remained steady for all four groups, which indicated a shift from feedback to feedforward control as the target speed increased. The feedback-feedforward trade-off induced by stroke, aging and speed might be explained by a change in the transmission delay and neuromotor noise. The findings in this study improve our understanding of the mechanism underlying the sensorimotor control and neurological changes caused by stroke and aging.

## Introduction

Target-directed arm movements are essential components of people’s daily activities, which usually require high levels of motion speed and accuracy [1,2]. In general, the following are three sequential steps underlying the generation of target-directed arm movements: movement perception, movement programming and movement execution. Movement perception is a process in which the central nervous system (CNS) perceives the environment, including both the target location and terminal position from receptors; movement programming means generating motor commands to achieve the terminal accuracy based on task constraints. Finally, movement execution refers to motor commands being conveyed to effectors, where the goal-directed motions are actually conducted [3]. Most optimal motor behaviors reflect a combination of two interacting strategies, feedback and feedforward control, while target-directed arm movements belong to this category [4]. Feedback control is an essential cognitive and motor skill for people to optimize motor performance, which refers to a modification of movement with the sensory information, involving error detection and correction during target-directed movements. This control strategy can contribute to a high degree of terminal accuracy, but it also needs to account for the feedback loop delay [5]. Feedforward control is driven by the predictive efferent estimation process without sensory feedback; therefore, there is no delay in the feedback loop [1]. Many researchers have suggested that there is a hybrid of feedback and feedforward control rather than isolated feedback or feedforward sensorimotor control of human movements [6–9].

Target-directed tasks have been adopted in many previous studies to investigate sensorimotor control. Compared with stationary targets, the movements with ever-changing targets introduced more complex and difficult control schemes that receive substantial attention because the target positions that change over time may result in additional error messages between the positions of both the limb and target, and the CNS must plan and generate motor commands in a limited time period [6,10–15]. Reed et al. designed a series of horizontal tracking tasks with different vertical separations between the guiding target and the movement cursor, and they reported a reduction in online feedback control when the spatial separation of visual cues increased [11]. Sosnoff et al. [15] and Baweja et al. [12] detected the effects of the level and frequency of display information on feedback control during force tracking tasks. Huang and Hwang explored the control pathway during tracking motions with or without visual information [13]. Gritsenko et al. [6] and Lee et al. [14] investigated the ability of adaption to the disturbance of the target jump or rotation during reaching tasks and confirmed the integration of both feedforward and feedback control. The relationship between external factors, such as the target size [16], external force field [17], task orientation [18], and movement performance is important to understanding the underlying sensorimotor control. Movement speed, another external factor, could also influence the control strategies during target-directed movements. Gerisch et al. found a speed-related component in a model of the terminal accuracy in tracking tasks with continuous random targets [19]. Shin et al. observed declines in accuracy index (AI) with an increase in the movement frequency during finger tracking movements [20]. Maill et al. observed the tracking performance for normal individuals at the five target speeds and confirmed that the performance decrease with increasing target speed after the analysis of spectral compositions of the movement signals [21]. From previous studies, feedback component of sensorimotor control were affected by the external factors [19,21].

In addition to the external factors, the internal changes in the neurological structure caused by diseases, such as Tourette syndrome [22], Parkinson's disease [23], Huntington’s disease [24], stroke[25], and aging [26], could interfere with the sensorimotor control. Following a stroke, patients always have various degrees of cognitive and sensory impairments, which lead to a decreased perceptual motor function [27,28]. Over the past two decades, tasks involved with target-directed movements were utilized to investigate the sensorimotor dysfunction of patients after stroke [25,29,30]. Carey et al. designed finger tracking tasks for patients after stroke with different levels of information programming and revealed that stroke-induced sensorimotor impairment could result in less tracking accuracy in the condition of the stimulus-response compatibility [29]. Cirtea et al. evaluated stroke-induced cognitive impairment on the capacity for motor learning through reaching tasks with two types of feedback conditions, which were terminal feedback and concurrent feedback [30]. Based on Fitts’ law, McCrea and Eng found that the greater neuromotor noise caused by stroke influenced motor planning during reaching tasks [25]. Rohrer et al. depicted less fragmented and more coordinated reaching movements for stroke survivors during recovery, which might result from the improvement in the sensorimotor control ability [31]. Also, age-related changes in the sensorimotor control have been reported. Bennett et al. suggested that the elderly individuals exhibited strategic changes in movement kinematics when aiming at vertically located targets [26], while Hegele et al. illustrated that younger subjects showed superior explicit knowledge of adaptation to novel visuomotor rotations and gains during pointing tasks [32]. On account of the neurological damages or aging, subjects have been supposed to rely more on feedback control during tracking movements [29,33], reaching movements [25,31,34].

Although many studies have been devoted to either external factors or an internal neurological structure with sensorimotor control, the combination of target speed and internal neurological damages on sensorimotor control was seldom investigated. Although the shift from feedback to feedforward in motor control as the movement speed increases has been reported by previous studies [6,19,35], it is an innovative view to indicate the change in the motor control strategy based on both temporal and frequency characteristics. Therefore, the purpose of this study focused on how stroke and aging influence sensorimotor control, and we selected a series of elbow tracking tasks with 6 uniformly changing speeds, which was performed by stroke patients and healthy subjects (young and age-matched controls). We hypothesized that stroke- and ageing-induced neurological change as well as target speed would affect sensorimotor control. Three representative indicators of movement performance, which were extracted from the tracking trajectories in the both the time and frequency domains, were used to understand the way that the subjects responded to changing-targets at various speeds and to investigate the stroke- and age-induced change in sensorimotor control.

## Materials and Methods

### Subjects

A total of 31 subjects were recruited in this study. There were 11 chronic stroke patients in the study group (9 males and 2 females, mean age: 47±10.86 years), ten age-matched healthy controls (5 males and 5 females, mean age: 51±6.24 years) and ten healthy young controls (5 males and 5 females, mean age: 22±1.63 years), respectively. Table 1 summarizes the basic information of the patients after stroke. The following criteria should be followed for the patient group: (1) unilateral lesion with onset at least 1 year before the experiments were performed; (2) satisfactory vision and mental capacity to follow directions and perform experimental procedures; (3) voluntarily flex/extension of the elbow between 30 and 90 degrees on the affected sides; and (4) ability to provide informed consent. The human ethical committee of the Sun Yat-sen University and Hong Kong Polytechnic University approved the experiment in this study. Before the experiment, the experimental protocols were introduced to all the subjects and the written informed consents of all the subjects were provided to participate in this study and publish these case details.

**Table 1:** Basic data of eleven subjects after stroke.

Subject	Age	Sex	Lesion side	Type of stroke	Years after stroke	Modified Ashworth scale
**Subject 1**	49	M	L	Isch	1 yr	1+
**Subject 2**	42	M	L	Hemo	4 yrs	1+
**Subject 3**	57	M	R	Isch	13 yrs	3
**Subject 4**	52	M	R	Hemo	4 yrs	2
**Subject 5**	39	M	R	Hemo	11 yrs	2
**Subject 6**	60	M	R	Isch	5 yrs	1
**Subject 7**	46	M	R	Isch	5 yrs	1+
**Subject 8**	46	F	L	Isch	2 yrs	1
**Subject 9**	51	F	L	Isch	1 yr	1+
**Subject 10**	57	M	R	Isch	3yrs	1
**Subject 11**	21	M	R	Hemo	4 yrs	1+

Abbreviations: F, female; M, male; R, right; L, left; Isch, ischemic stroke; Hemo, hemorrhagic stroke. Modified Ashworth scale: 0 = no increase in tone; 1 = slight increase in muscle tone; 1+ = slight increase in muscle tone, manifested by a catch, followed by minimal resistance throughout the remainder; 2 = more marked increase in muscle tone through most of ROM, but affected part move easily; 3 = considerable increase in muscle tone, passive movement difficult; 4 = affected part rigid.

### Apparatus

**Fig 1:**
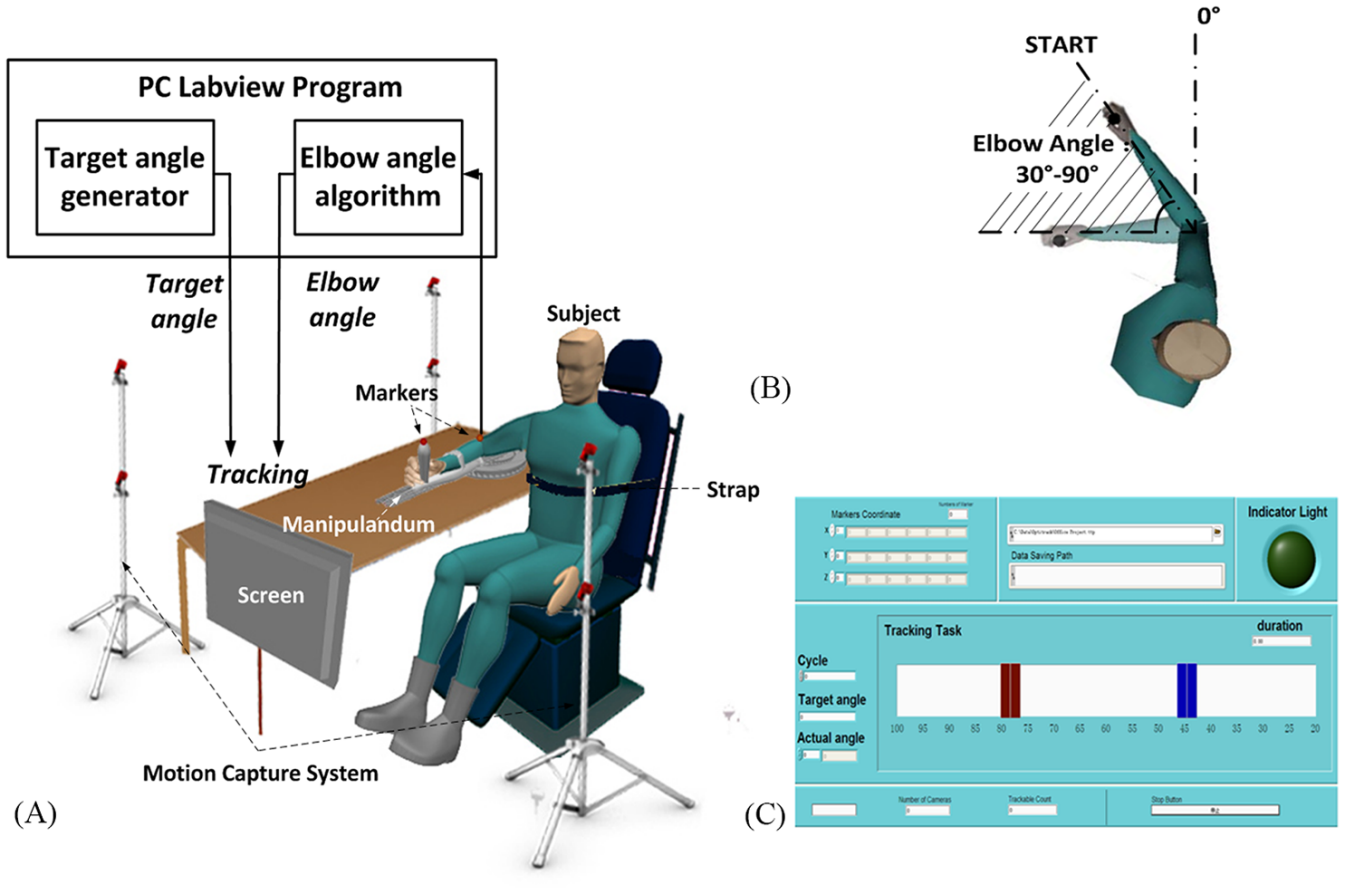
Experimental setup and conditions. (a) a schematic drawing of the experimental setup and the control system. (b) Diagrammatic representations of the range of the elbow angle during the experimental: shaded area illustrated that the range of the elbow flexion and extension was 30 degree to 90 degree, while the straight arm was at 0 degree. (c) The interactive interface of the tracking tasks: both the tracking target (the blue slider) and the actual elbow angle (the red slider) moved horizontally.

As shown in Fig 1A, the subjects were seated on a comfortable chair with a screen in front of the subjects. Each patient’s shoulder was at approximately 90 degrees of abduction, and the torso was attached to the back of the chair. The actual elbow and target angles were displayed with real-time visual feedback on the screen. The target on the screen was a blue slider that was 1×3 cm in size, while the actual angle of elbow joint was shown as a same-sized red slider that could be watched clearly (Fig 1C). The subjects were instructed to attach their forearms to a light aluminum manipulandum with their hands gripping the handle at the end of the manipulandum. The manipulandum for the tracking task was custom-designed and the rotation axis of it was connected with a ball bearing that could support elbow flexion and extension with negligible friction torque. During the elbow movements for young and age-matched controls, the coordinates of the markers attached to the handle and elbow joint were captured by a motion capture system at a sampling frequency of 100 Hz (NATURALPOINT, OPTITRACK, USA) and converted into real-time angular displacement using the Labview program. As for the patients, a flexible electrogoniometer (PENNY & GILES, UK) attached to the manipulandum was used to measure the actual elbow angle [36].

### Tracking procedure

The trajectories of the tracking target were designed as sinusoids to simulate the bell-shape velocity profile of daily single-joint movement, and all of the subjects were required to track the target by performing elbow flexion and extension between 30 and 90 degrees for 36 seconds in each trial (shown in Fig 1B). The frequency of each trial was selected from one of six levels, which ranged from 1/12 Hz to 1/2 Hz with a step of 1/12 Hz. The maximum tracking angular speeds were 15.7, 31.4, 47.1, 62.8, 78.5, and 94.2 deg/s, respectively, which covered the normal speed range of human daily movements and were comfortable for most post-stroke patients to follow. During the experiment, the subjects conducted a total of 18 trials after 4–5 practice trials that were divided into 3 blocks. There were 6 trials corresponding to 6 velocities, respectively, in each block, and the sequence of six speeds was randomly arranged. After a random delay of 2 to 5 seconds, the target slider started moving and the subjects began to control their elbows to follow the target slider as accurately as possible. For post-stroke patients, tracking tasks were conducted by both the affected and unaffected sides, and for young and age-matched controls, tracking tasks were only performed with their dominant sides.

### Outcome measures

To extract the actual angular signals from the raw records, a fourth-order Butterworth filter with a cut-off frequency of 20 Hz was applied. The tracking performance was evaluated in both the time and frequency domains. In the time domain, both the root mean square error (RMSE) and the normalized integrated jerk (NIJ) of the tracking movements were measured, while in the frequency domain, an integral of the power spectrum density of normalized speed (IPNS) was applied.

The tracking error, which is the separation between the targeted and actual trajectory, was characterized by the root mean square error (RMSE) [37] in this study. The RMSE can be calculated as follows:

$$RMSE=\sqrt{\sum_{i=1}^{N}{\left(\theta_{act}(i)-\theta_{tar}(i)\right)}^{2}/N}$$ (1)

Where *θ*_*act*_(*i*) and *θ*_*tar*_(*i*) represent the actual and target elbow angles at the i-th sample, respectively. And N is the total number of samples of the tracking trajecory in a trial.

Jerk-based indexes are empirical measurements of movement smoothness and smaller values of jerk-based indexes always refer to smoother and less fragmented movement [38]. The normalized integrated jerk (NIJ) was utilized in this study, which is summarized as follows [39]:

$$NIJ=\sqrt{{\text{duration}}^{\text{5}}/2length^{2}\times \int_{tstart}^{tend}jerk^{2}(t)dt}$$ (2)

Where *erk = d* ^*3*^ *θ/dt* ^*3*^, *θ*(*t*) is the angular displacement of elbow; *tstart* and *tend* indicate the starting and ending time points of the movement trajectory; and *duration* ^*5*^ */2length* ^*2*^ is a normalized factor proposed by Teulings et al. [40] In this study, each elbow flexion- extension cycle was divided into a flexion phase and an extension phase, and the mean values of the NIJ in all monotonous flexion or extension phases were calculated.

For each trial, the angular speed signals derived from the elbow angle were filtered by a 4-order low-pass Butterworth filter with a cut-off frequency of 20Hz, and the power spectrum of the angular speed signals for each 36-s dataset was obtained with fast Fourier transform. There is a high-frequency component in the power spectrum in addition to the low-frequency target component. Miall et al. reported that the high-frequency component within 0.8–1.8Hz in the power spectrum can indicate the nature of the human sensorimotor feedback [21,41]. Therefore, the integral of the power spectrum density of normalized speed (IPNS) in the frequency band of 0.8–1.8Hz can be calculated as [21]:

$$IPNS=\sum_{i=0.8}^{1.8}p_{i}$$ (3)

Where *i* refers to the sampling point of the frequency value, which was ranges from 0.8 to 1.8 Hz and *p*_*i*_ refers to the power density spectrum value of normalized speed at the frequency of *i*.

### Statistical analysis

To test the main effect of group (the affected sides of stroke patients, unaffected sides of stroke patients, age-matched controls and young controls), tracking speed (V1: 15.7, V2:31.4, V3:47.1, V4:62.8, V5:78.5, and V6:94.2 deg/s) and the interaction effect of both the two factors on the RMSE, NIJ and IPNS values, two-way ANOVA was utilized. The paired t-test (two-tailed) was applied to analyze the variance of RMSE, NIJ and IPNS between the affected and unaffected sides at the same speed and the difference of the RMSE, NIJ and IPNS between the two successive tracking speeds in the same group. In the following, post hoc Bonferroni’s multiple comparison tests (correction of alpha was 3) were performed to determine the difference of the RMSE, NIJ and IPNS among the three groups (affected sides or unaffected sides, age-matched controls and young controls). The significance level was set at 0.05 for all statistical analyses, which were performed using SPSS 19. (SPSS Inc., CHICAGO, ILLINOIS, USA).

## Results

### Root mean square error (RMSE)

**Fig 2:**
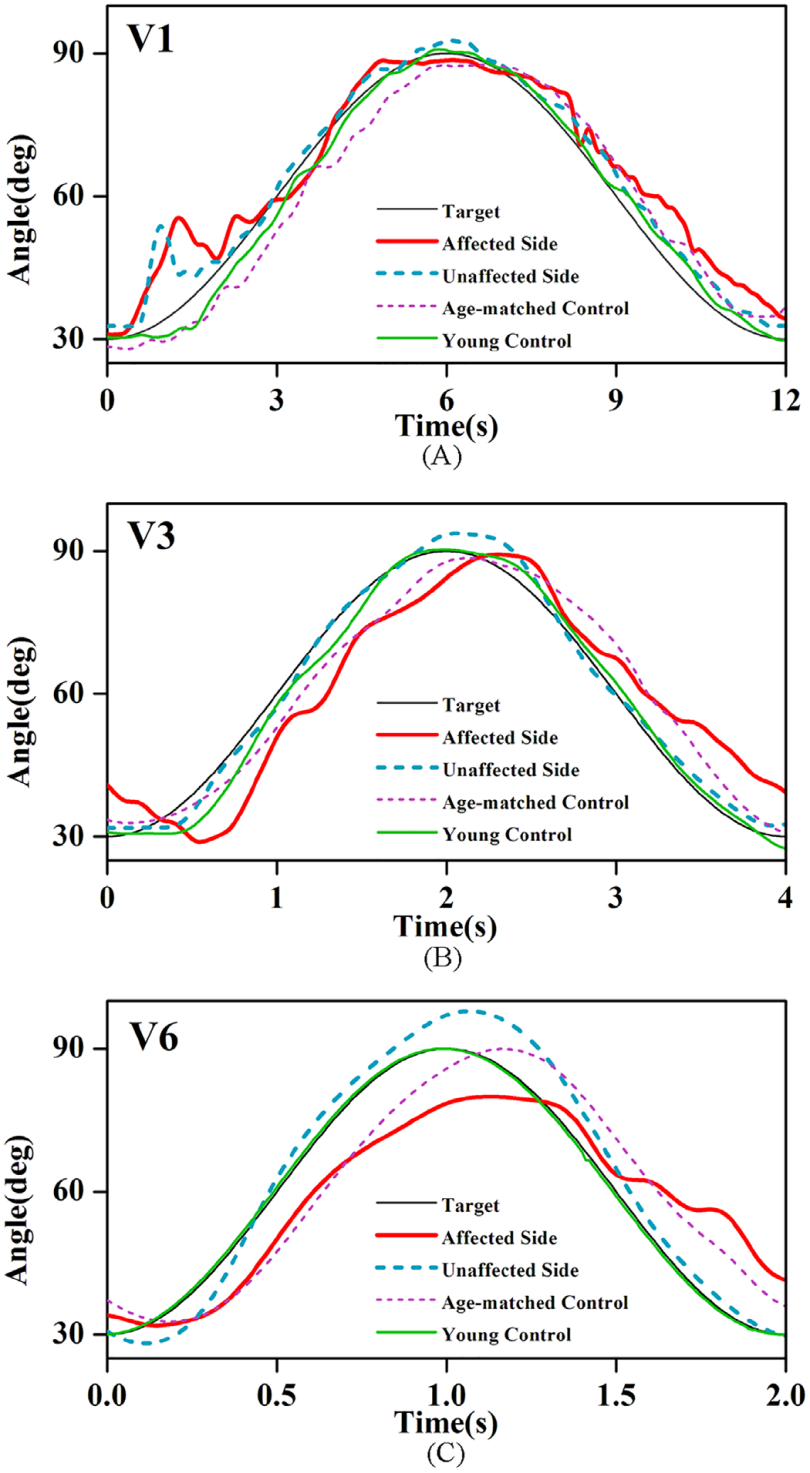
The actual elbow and the target angles in four groups at different speeds. The angles at (A) V1 = 15.7deg/s, (B) V3 = 47.1deg/s and (C) V6 = 94.2deg/s for the affected side and unaffected side of a patient after stroke, an age-matched control and a young control.

Fig 2A–2C illustrate the typical elbow angular signals of the affected and unaffected sides of a stroke patient, an age-matched control and a young control in a cycle at the speeds of V1, V3 and V6. We found that the actual elbow trajectories of all of the groups fluctuated with the changing-targets, whereas there were varying degrees of errors between the elbow and the target positions.

**Fig 3:**
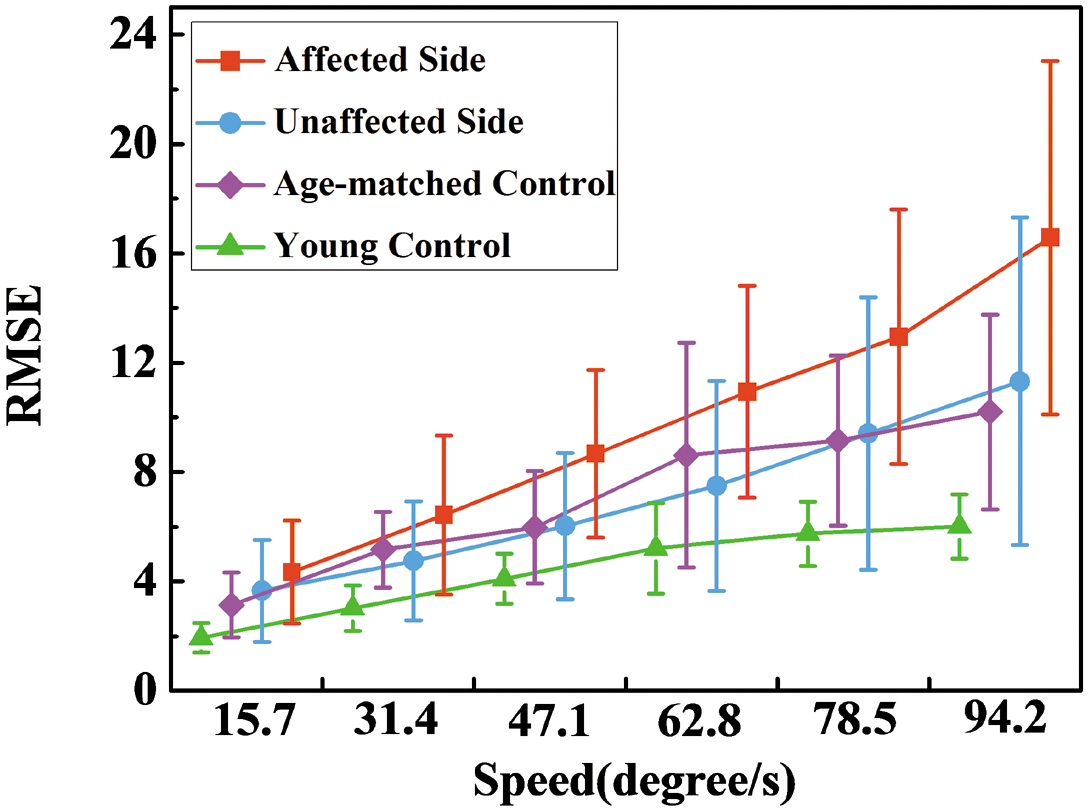
The RMSE in four groups at 6 tracking speeds. RMSE refers to the Root mean squre error.

Fig 3 demonstrates the mean values of RMSE in each group across 6 tracking speeds. Both the factors of group (P<0.01, F = 20.90) and tracking speed (P<0.01, F = 21.06) showed notable influences on the RMSE. There was an increase in the RMSE for all the four groups as the tracking speed increased. Paired t-tests revealed that for both the two sides of the patients after stroke, there was a significant rise between each two successive speeds (P<0.01). As for the age-matched controls, the differences between V1 and V2 (P<0.01), V3 and V4 (P<0.01), V5 and V6 (P = 0.042) were significant, while as for the young normal controls, the variances between V1 and V2 (P<0.01), V2 and V3 (P<0.01), V3 and V4 (P<0.01) were remarkable. Moreover, in comparison with unaffected sides, the affected sides showed significant larger values of RMSE at all 6 speeds (P<0.01). According to the results of multiple comparisons, both the two normal groups had significant smaller values of RMSE than the affected sides of stroke patients (P<0.01), while only the young controls depicted notable smaller RMSE than the unaffected sides (P<0.01). There was also a significant interaction between two factors of group and speed on the RMSE values (P = 0.033, F = 1.82).

### Normalized integrated jerk (NIJ)

**Fig 4:**
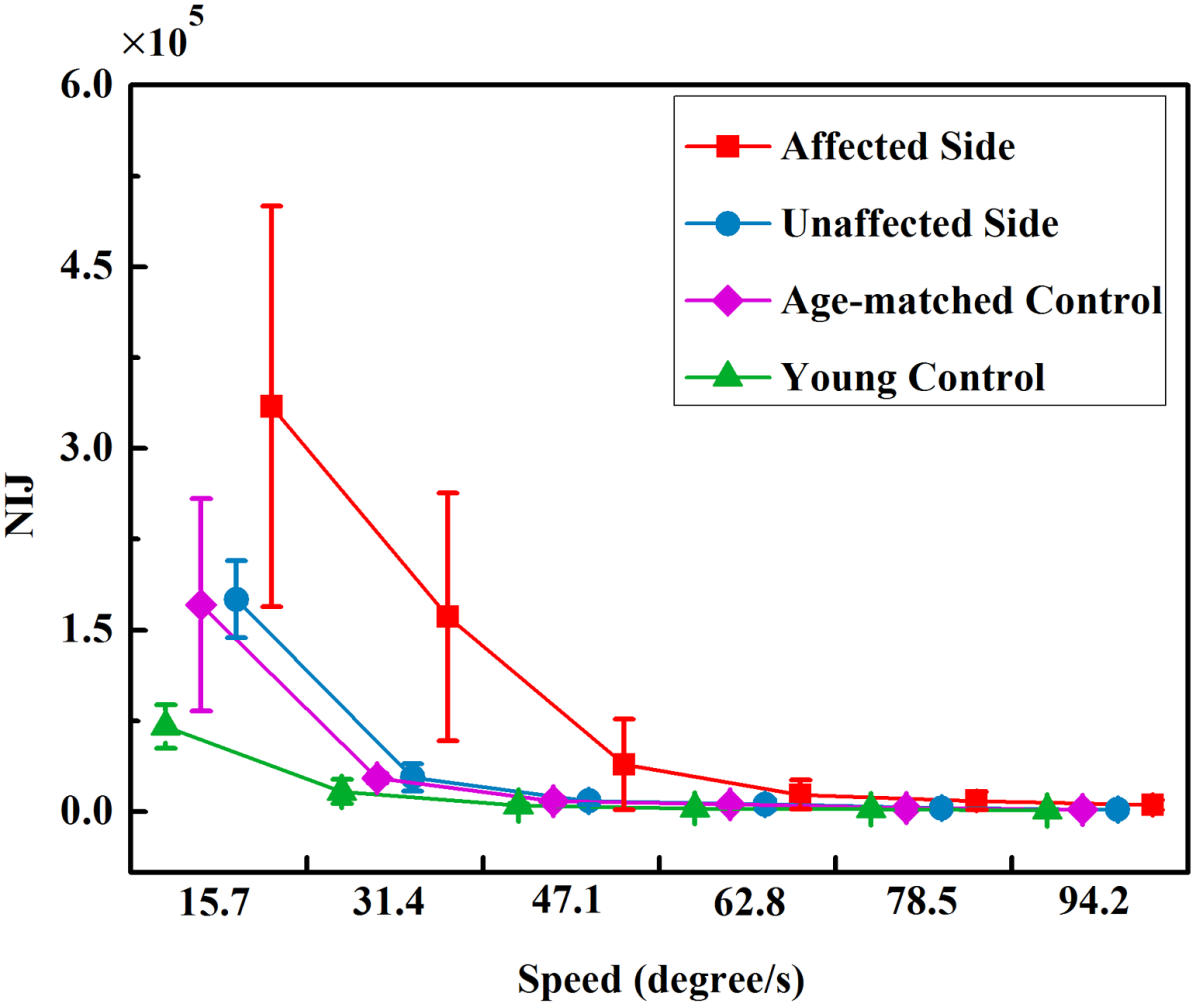
The NIJ in four groups at 6 tracking speeds. NIJ refers to the Normalized integrated jerk.

As shown in Fig 2, it is noticed that the actual elbow trajectories changed discontinuously for all the groups. The mean values of the NIJ in each group across 6 tracking speeds were calculated and depicted in Fig 4. Two-way ANOVA revealed that there were significant impacts of both the group (P<0.01, F = 27.39) and tracking speed (P<0.01, F = 85.12) on the NIJ. The average NIJ values showed the following order at 6 speeds: young controls < age-matched controls < unaffected sides of stroke patients < affected sides of stroke patients. Paired t-tests suggested that the affected sides had significantly larger NIJ values than the unaffected sides across all 6 speeds (P<0.01). The NIJ values from two control groups were remarkably smaller than those from the affected sides of stroke patients at all 6 speeds according to the post hoc analysis (P<0.01). Between young and aged healthy individuals, the NIJ values of age-matched controls were significantly higher than those of the young controls (P = 0.011). For all four groups, Paired t-tests revealed that the NIJ values tended to significant declines from V1 to V2 (all groups: P<0.01) and from V2 to V3 (affected sides: P = 0.027; unaffected sides: P<0.01; age-matched controls: P<0.01; young controls: P = 0.042), and there was a non-significant decrease between other successive speeds (V3 toV4, V4 to V5 and V5 to V6). Moreover, the interaction analysis indicated that there was significant interaction between the factors of group and speed in the NIJ values (P<0.01, F = 8.349).

### Integral of the power spectrum of the normalized speed (IPNS)

**Fig 5:**
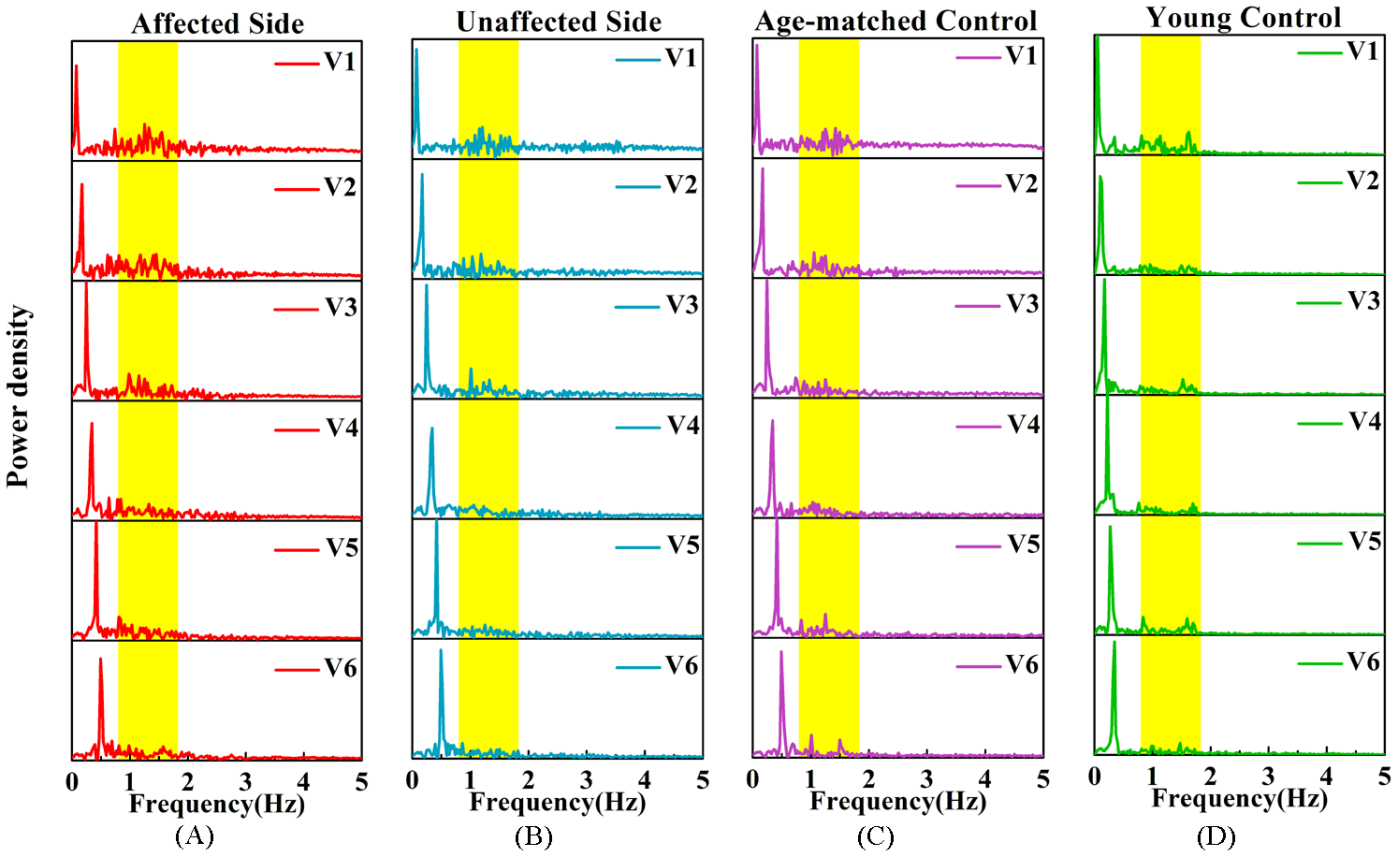
The power spectrum of the normalized speed of four groups at different speeds. The power spectrum of the normalized speed across 6 tracking speeds (V1-V6) for (A) affected side of a patient after stroke, (B) unaffected side of a patient after stroke, (C) an old control and (D) a young control; the yellow bars highlight frequency range of intermittent movements (0.8–1.8 Hz).

Fig 5A–5D demonstrate the power spectrum of the normalized speed of the affected and unaffected sides of a stroke patient, an age-matched and a young healthy subject under 6 tracking speeds respectively. In addition to the component of the low frequency corresponding to the target movements, there was a relatively high-frequency component within 0.8–1.8Hz in each power density spectrum, and it is also illustrated from Fig 5 that the amplitudes of the power density spectrum within 0.8–1.8 Hz were relatively larger at a lower tracking speed.

**Fig 6:**
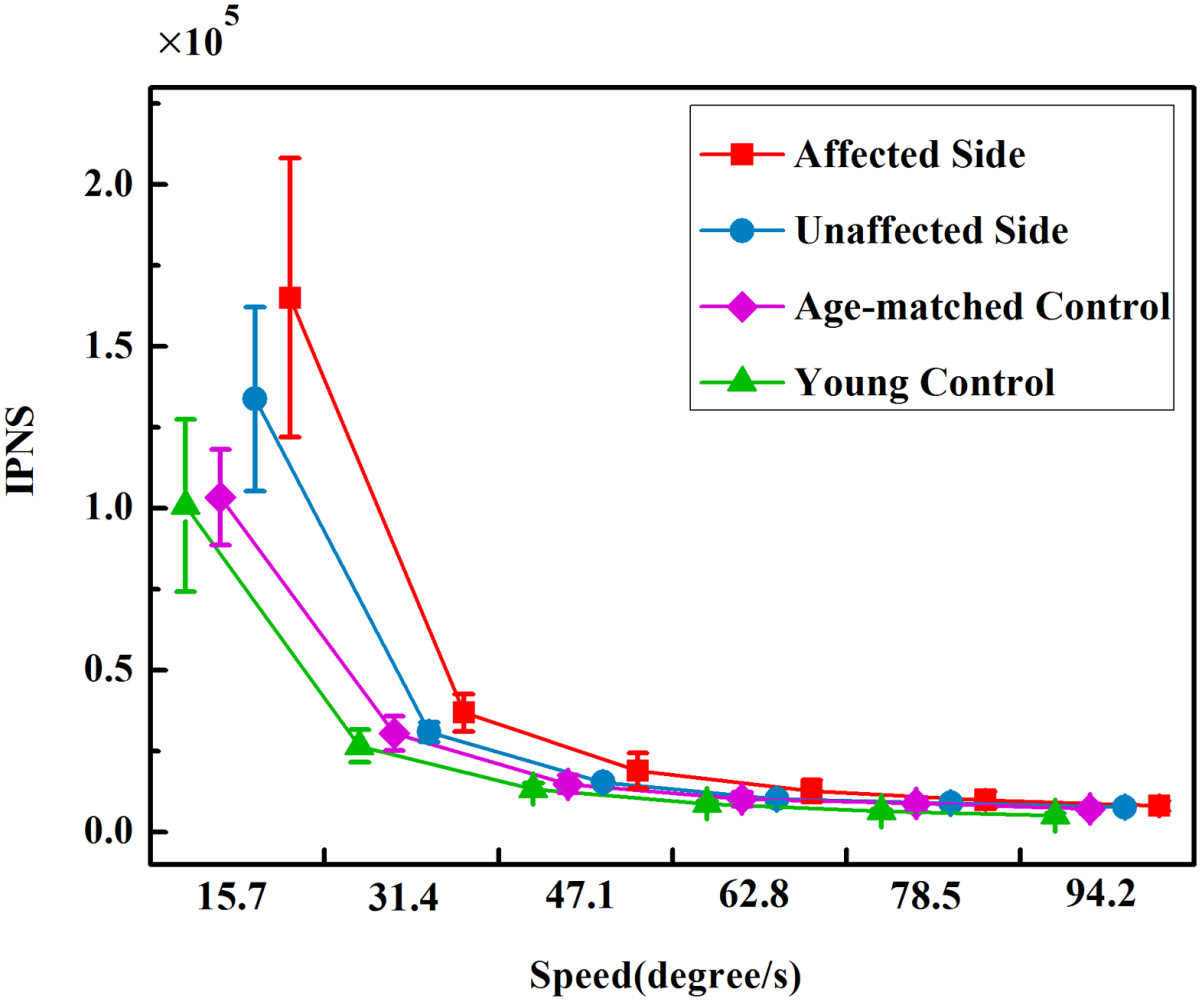
The IPNS in four groups at 6 tracking speeds. IPNS refers to Integral of the power spectrum of the normalized speed.

Fig 6 quantifies the mean values of the IPNS in each group at 6 tracking speeds. Two-way ANOVA found that both the group (P<0.01, F = 16.77) and tracking speed (P<0.01, F = 556.26) had a significant influence on the IPNS. The IPNS values of the four groups had the following order: young controls < age-matched controls < unaffected sides of stroke patients < affected sides of stroke patients. Between groups, paired t-tests revealed that there were significant increases in the IPNS values of the unaffected sides compared with those of the affected sides (P<0.01). Post hoc analyses revealed that the IPNS values of young controls were dramatically smaller than those of the affected sides across 6 speeds (P<0.01). Moreover, there were relatively sharp declines in the IPNS values from V1 to V3 and stable IPNS values from V4 to V6. According to the paired t-tests, the decreases were significant from V1 to V2 (all groups: P<0.01), from V2 to V3 (all groups: P<0.01), and there was a non-significant difference between two successive speeds when the tracking speed was greater than V3 (V3 toV4, V4 to V5 and V5 to V6) in all of the groups. Additionally, a significant interaction between the factors of group and speed in the IPNS values was indicated by the interaction analysis (P<0.01, F = 8.994).

## Discussion

In this study, we investigated the changes in the sensorimotor control strategy based on the both temporal and frequency characteristics of the tracking performance, and significant influences of target speed and internal neurological damages (stroke and aging), on the outcome measures (RMSE, NIJ and IPNS) were demonstrated.

### Representation, characterization and internal mechanism of feedback control

The discontinuity in target-directed behaviors can be called as intermittency, which is considered a hallmark of the feedback component of sensorimotor control [21,42]. In this study, intermittency can be observed in elbow trajectories during the tracking tasks in Fig 2, which is in agreement with the findings in the previous tracking studies [9,21]. Jerk-based indexes reflected movement smoothness and were used to evaluate the intermittency of movements in visual tracking or reaching tasks [31,37,43]. However, jerk-based measures, such as integrated squared jerk (ISJ) and root mean squared jerk (RMSJ), always rely on movement speed; therefore, normalization is needed to achieve intermittency analysis among different speeds. Another way to represent the intermittency of movements was to derive the feedback component from the power spectrum of the speed trajectories [42,44]. Miall et al. suggested that the intermittent tracking responses between 0.8–1.8 Hz are indicative of the incorporation of error-magnitude dependent process and time dependent process caused by delays in the visual feedback pathways [21]. It has been reported that the power spectrum values of the speed trajectories in the similar frequency range during visual tracking are larger than those of non-visual tracking movements [21].

The intermittency in the tracking movements, could be the comprehensive results of the innate features of feedback control loop, which mainly include transmission delay and neuromotor noise [4,13]. Feedback control relies on the sensory information of both the desired goals and terminal performance which are sampled from receptors [21]. During the process of closed-loop feedback control, the central nervous system generates a feedback motor command to correct the distance error between the actual position of the terminal and desired target immediately after the error is detected. The sampling rate of the input information flow has been proved to be as high as 500-10000Hz [35]. The transmission delay in the nervous system is primarily due to the finite axonal conduction speed and distance between neurons [45], and it always gives rise to a misalignment of the position between the moving limb and ever-changing target. Previous studies have suggested that inevitable delays in the visual-motor feedback loop could determine the number and frequency of error-based corrective submovements during the tracking tasks and the motor performances were limited by introducing the external delay of the joystick position which could help elucidate the effect of a delay in the internal process of the neural network [41,44]. On the other hand, there are numerous explanations that human neural pathways are like noisy information processing channels in which signals are contaminated by neuromotor noise [46]. During target-directed movements, the increase in the neuromotor noise primarily affects the motor programming and motor execution during target-directed movements [25]. Neuromotor noise could corrupt both the input terminal information and the target information, and may create larger stochastic-based disparities between the planned and the desired positions, resulting in submovements in a relatively higher frequency to hit the target [47,48].

### Effect of the target speed on the feedback–feedforward control trade-off

The RMSE values increased significantly as the target speed increases, while the NIJ and IPNS values were higher when the tracking speed was below 47.1 deg/s, and neither the NIJ nor IPNS changed much when the tracking speed was higher than 47.1 deg/s in all four groups. This finding can be explained by a shift from feedback control to feedforward control as the target speed increased as well as an ‘upper threshold of speed’ beyond which all groups almost depended on feedforward control for motor control. From the view of control, long delays in feedback are supposed to have negative effects of the real-time control performance, such as system stability [49]. The transmission delay in the feedback control loop can be negligible in the control of slow movements whereas it makes up a large proportion of movement duration during fast movements [35]. During the rapid movements, integration of the sensory information and generation of motor commands over a short period require the nervous system, which might exceed its maximum capability [35]. Furthermore, Guigon et al. suggested that faster movements require larger motor commands which might introduce neuromotor noise with a larger amplitude and higher frequency [50]. Harris and Wolpert revealed that slow movements operated by control signals with a relatively small size, and the noise in neural command was signal-dependent [51]. The large-amplitude and high-frequency neuromotor noise in fast movement may generate great error which may also exceed the upper tolerance of the motor control system [52]. Therefore, feedforward control, which is an open control loop with a negligible delay, is preferred. The nervous system itself can predict the behaviors according to the motor commands; and then, the error between the predicted limb position and target location is used to generate new commands. The duration of the feedforward loop is small, and the input signal copies motor commands accompanied by a low degree of noise [1]. A computational internal model was presented by Wolpert and Kawatoto to express the complex processes of motor control by the cerebellum [53]. The model consisted of an inverse model that refers to the feedback process and a forward model that refers to the feedforward process. The mathematical model that considers noise and delay suggested that feedforward control plays a more important role in fast movements [52]. Therefore, based on the findings reported in previous studies, a shift from feedback to feedforward control could explained the decrease in the NIJ and IPNS values as the target speed increased, which might be due to relatively longer transmission delay and larger neuromotor noise in the faster movements.

### Effects of stroke and aging on the feedback–feedforword control trade-off

The higher RMSE, NIJ and IPNS values of the affected sides compared with those of the age-matched controls illustrated that the post-stroke patients relied more on feedback control than normal individuals, while the larger RMSE, NIJ and IPNS values of the age-matched controls compared with the young controls also reflects more reliance on the feedback control induced by aging. The different patterns of the motor control strategy induced by stroke and aging have been assumed to result from an increase in the transmission delays and neuromotor noise in the neuromuscular system. The longer reaction time of stroke patients [54] and elderly individuals [55] has been reported in the goal-directed aiming tasks which may reflect longer transmission delays in the visual feedback loop. Additionally, McCrea and Eng found that the consequences of stroke were greater neuromotor noise by deriving the slope of Fitts’ law during reaching movements with various target sizes and distances [25], while Reinkensmeyer et al. used population vector coding models to demonstrate that the loss of neurons and impairments of several motor areas in stroke survivors would add more noise to motor commands [56]. Beer et al. also held the view that the increase in the neuromotor noise, which lead to stroke patients caused by abnormal background motor neuron activity and co-contraction [3]. Potson et al. suggested that aging is related to an increase in the amplitude of neuromotor noise [57], and Pohl et al. also agreed that there are central processing deficits in aged individuals, such as increasing neuromotor noise, which lead to more discrete adjustments in the trajectories [55]. The greater neuromotor noise in stroke patients and ageing individuals needs more feedback component for adjustment [58]. Furthermore, spasticity, abnormal synergy, weakness in the musculoskeletal system, and the increased joint visco-elasticity in patients after stroke [58,59] may allow the limb to move off the planned trajectory, requiring more subconsciously corrected movements for compensation through feedback.

## Conclusions

In this study, voluntary elbow tracking tasks were used to investigate the stroke- and age- induced changes in the sensorimotor control strategies at various target speeds. According to the temporal and frequency analysis of the tracking performance, both the external factor (speed) and internal neurological damages (stroke and aging) influence the feedback-feedforward trade-off. The findings in this study could help elucidate the mechanism underlying sensorimotor control changes that are due to stroke, aging and speed as well as provide evidence that selection of the target speed, which affects the motor control strategy, should be considered when designing visually target-directed tasks for clinical evaluation or purposeful interventions for stroke rehabilitation. For instance, higher speed may be suitable for the evaluation of feedforward control, and lower speed should be adopted to evaluate the sensorimotor control with more feedback component. In the future work, a larger sample size with chronic and acute stroke and controlled for lesion location should be employed to further explore the potential clinical effectiveness.
